# Age- and Sex-Dependent Interpretation of C-Reactive Protein Cutoffs: A Sixteen-Year Large-Scale Clinical Laboratory Data Analysis

**DOI:** 10.3390/diagnostics16091268

**Published:** 2026-04-23

**Authors:** Jeong Su Han, Jae-Sik Jeon, Bo Kyeung Jung, Jae Kyung Kim

**Affiliations:** 1 Department of Biomedical Laboratory Science, College of Health Sciences, Dankook University, Cheonan-si 31116, Republic of Korea; jshan1162@naver.com (J.S.H.); zenty87@naver.com (J.-S.J.); 2 Department of Laboratory Medicine, College of Medicine, Dankook University, Cheonan-si 31116, Republic of Korea; lovegodmother@hanmail.net

**Keywords:** age–sex stratification, biomarkers, C-reactive protein, cutoff interpretation, inflammation, retrospective studies

## Abstract

**Background/Objectives:** The clinical meaning of a given C-reactive protein (CRP) threshold may differ by age and sex; however, a comprehensive framework to elucidate demographic differences is lacking. We examined age- and sex-related differences in the central tendency and upper tail of the CRP distribution and their implications for fixed-threshold interpretation. **Methods:** We retrospectively analyzed 1,845,151 serum CRP results from 336,360 individuals at a single tertiary-care hospital. Quantile regression estimated the median (q = 0.50) and 95th percentile (q = 0.95), and logistic regression assessed frequencies and odds ratios (ORs) for CRP thresholds ≥1, ≥3, and ≥10 mg/dL. Patient-year-first sensitivity and generalized estimating equation (GEE) analyses were performed. **Results:** CRP showed marked right-skewness, with a progressively heavier upper tail with age. The median increased from 0.19/0.26 mg/dL in females/males aged < 1 year to 2.55/3.44 mg/dL in those aged ≥ 85 years. The 95th percentile increased from 3.28/4.31 to 17.50/18.80 mg/dL. Among records aged ≥ 85 years, CRPs ≥ 1, ≥3, and ≥10 mg/dL occurred in 67.6%/72.3%, 46.6%/53.4%, and 15.3%/19.1% of females/males, respectively. For CRP ≥ 10 mg/dL, ORs increased stepwise to 12.7, 15.4, and 18.1 in those aged 65–74, 75–84, and ≥85 years, respectively. These patterns were preserved in sensitivity and GEE analyses. **Conclusions:** CRP distributions differed substantially by age and sex, indicating that a single threshold may not have uniform interpretive meaning across demographic groups. These findings support more context-aware interpretation of CRP thresholds in hospital-based practice, while suggesting that observed differences reflect not only demographic variation but also differences in underlying case-mix and clinical complexity.

## 1. Introduction

C-reactive protein (CRP) is one of the most widely used acute-phase reactants in clinical practice and serves as a representative biomarker of systemic inflammatory burden in a broad range of conditions, including infection, tissue injury, autoimmune disease, malignancy, and postoperative states [1]. Owing to its high accessibility and relatively straightforward interpretation, CRP is widely used as a practical tool for assessing the presence and degree of inflammation across diverse clinical settings, including outpatient clinics, emergency departments, and inpatient care [2]. In practice, CRP is used not merely as an ancillary laboratory value, but also to support a variety of clinical decisions, such as whether additional diagnostic evaluation is needed, whether antimicrobial therapy should be initiated, how the disease course should be monitored, and how severity should be assessed [3].

Despite its broad clinical utility, CRP is commonly interpreted on the basis of absolute values, with routine categorization into normal versus elevated levels or into mild, moderate, and marked elevation according to predefined thresholds [4]. Although this approach is convenient, it carries the implicit assumption that the same CRP value has equivalent clinical meaning across different demographic groups. However, age and sex are key biological determinants that may influence the baseline level and overall distribution of inflammatory responses [5], yet these differences are often insufficiently incorporated into routine clinical interpretation [6]. Consequently, the same threshold may represent a relatively common value in one group but a clear abnormal signal in another, whereas actual interpretation often continues to rely on uniform criteria.

This issue is becoming increasingly critical in healthcare systems facing rapid population aging [7]. In older adults, a chronic low-grade inflammatory state, commonly referred to as inflammaging, has been described and may affect the baseline level and distribution of inflammatory biomarkers, including CRP [8]. In addition, the accumulation of comorbidities, functional decline, and repeated contact with healthcare services may further shape the CRP distribution in older individuals differently from that in younger populations [9]. Accordingly, CRP elevation in older patients should be interpreted not only in terms of the absolute value itself, but also with reference to its relative rarity within that age group, the distributional position and frequency of threshold exceedance, and its interpretive distinction from other age strata. As CRP is characterized by substantial distributional asymmetry and a clinically important upper tail, approaches that rely primarily on a single summary measure, such as the mean, may not provide sufficient information for meaningful interpretation [10]. In this context, quantifying age-related shifts in the 95th percentile is important not merely for describing extreme values, but also for evaluating how the boundary of what may be considered abnormal is itself reconfigured across age groups.

Previous studies have often focused on specific disease groups, limited age ranges, or cross-sectional designs, or have primarily emphasized central tendency, particularly the mean [11]. In hospital-based laboratory data, repeated measurements are common, and multiple test results from the same individual may influence the overall distribution [12]. Therefore, a comprehensive analytical framework is needed to understand demographic differences in CRP, incorporating not only central tendency but also the upper-tail distribution, threshold-based frequencies, and adjustment for repeated testing.

Against this background, we used a large CRP testing dataset accumulated at a single tertiary-care hospital (Dankook University Hospital, Cheonan, Republic of Korea) between 2008 and 2023 to evaluate how the location of the CRP distribution and the frequency of values exceeding clinically relevant thresholds (≥1, ≥3, and ≥10 mg/dL) vary according to age and sex. In addition to the distributional center (median), we quantified the clinically important upper tail (95th percentile) using quantile regression, compared threshold-based frequencies using logistic regression, and examined the robustness of the observed patterns through sensitivity analyses that accounted for the influence of repeated measurements. Through this approach, we aimed to show that the weight of an “abnormal signal” conveyed by a given cutoff may differ according to demographic context, and to provide a distribution-based interpretive framework that may be particularly useful for distinguishing between common mild elevation and relatively infrequent but clinically important marked elevation in older adults.

## 2. Materials and Methods

### 2.1. Study Design and Population

This was a retrospective observational study based on serum CRP test records obtained at Dankook University Hospital, a single tertiary-care hospital in Cheonan, Republic of Korea. The study period was defined as October 2008 to December 2023. The study population comprised all serum CRP test records generated by the hospital clinical laboratory during this period, and record selection was performed stepwise according to the study flowchart. Of the total test records (*N* = 1,845,824), 505 quality control (QC) results derived from non-patient specimens were excluded. An additional 61 non-analytical records that could not be standardized with respect to unit or code, as well as 107 records with missing age information, were also excluded. Consequently, 1,845,151 CRP test records were included in the final analysis. The primary analysis was conducted at the test-record level, whereas separate sensitivity analyses were performed to account for the potential within-patient correlation arising from repeated measurements. The study design and reporting followed the Strengthening the Reporting of Observational Studies in Epidemiology (STROBE) guidelines.

### 2.2. Data Collection and Variable Definitions

The dataset consisted of anonymized laboratory records extracted from the laboratory information system (LIS), and no personally identifiable information was included. The analytic variables were sex, age, and CRP concentration (mg/dL). Detailed linked clinical variables, such as diagnosis, comorbidity profile, care setting (outpatient, inpatient, or emergency department), medication use, smoking status, and body mass index, were not available in the analytic dataset. Accordingly, the present analysis was intended to characterize hospital-based CRP distributions across demographic strata rather than to fully disentangle the independent contribution of age from that of coexisting clinical complexity. In total, the analysis included test records generated from 336,360 patients. Age was calculated at the time of testing. Age groups were categorized into seven clinically relevant strata reflecting pediatric developmental stages and geriatric clinical stratification: <1 year, 1–12 years, 13–18 years, 19–64 years, 65–74 years, 75–84 years, and ≥85 years. Among older adults, individuals aged ≥65 years were further divided to reflect the clinical heterogeneity of the youngest-old, middle-old, and oldest-old subgroups [13,14].

The main outcomes were defined as the distributional characteristics of continuous CRP values, including the median and 95th percentile, and as elevated CRP based on predefined thresholds. Elevated CRP was dichotomized using three thresholds (≥1, ≥3, and ≥10 mg/dL [equivalent to ≥10, ≥30, and ≥100 mg/L]) to distinguish inflammatory levels with differing interpretive intensity. These thresholds were operationally defined to represent low-grade elevation, moderate-or-higher elevation, and marked inflammatory burden, respectively. To assess the influence of repeated measurements, the distribution of the number of CRP tests per patient was also summarized.

### 2.3. CRP Measurement

Serum CRP was measured by an automated immunoturbidimetric assay using the Cobas 8000 modular analyzer series (Roche Diagnostics, Mannheim, Germany) and the Tina-quant C-Reactive Protein Gen.3 reagent (Roche Diagnostics, Mannheim, Germany). Although the manufacturer specifies the analytical measurement range in mg/L, the LIS at our institution routinely reports CRP results in mg/dL. Accordingly, to preserve consistency with the original reporting format of the institutional LIS, all CRP values in this study were analyzed and reported in mg/dL. The basic reportable range was 0.03–35 mg/dL, and analytical precision was maintained at a coefficient of variation of <3%. For specimens exceeding the upper measurement limit, automated dilution and reanalysis procedures built into the analyzer were applied. For this study, the final result values stored in the LIS were used without modification.

After clot formation, specimens were centrifuged immediately to separate serum, stored at 2–8 °C, and analyzed within 24 h. In addition, internal QC and external quality assessment programs were continuously operated throughout the study period to ensure the consistency and reliability of analytical results across years.

### 2.4. Statistical Analysis

Differences in the CRP distribution according to age and sex were evaluated using quantile regression based on continuous test-level CRP values. The median (q = 0.50) and the 95th percentile (q = 0.95) were estimated to represent the distributional center and upper tail, respectively. The models included age group, sex, and the age group × sex interaction term. For categorical age-group models, coefficients were interpreted relative to the reference group of females aged <1 year. For ordinal age-trend models, coefficients represent per-category changes across ordered age groups, and the interaction term indicates sex-specific differences in the age gradient.

As repeated tests from the same patient were included, we considered the possibility that test results from patients with greater healthcare utilization and greater clinical instability could accumulate disproportionately in the dataset and thereby shift the CRP distribution upward. Such repeated measurements may allow certain patients to contribute disproportionately to the overall estimates and may weaken the assumption of independence among test-level observations. Therefore, as a sensitivity analysis to mitigate the influence of differential testing intensity and overrepresentation of frequently tested patients among frequently visiting patients, we additionally performed a patient-year-first analysis in which only the first CRP test for each patient in each calendar year was included. The “first test” for each year was defined according to the test date. Specifically, for each patient, the CRP result obtained on the earliest test date within the same year was selected as the representative value for that year. The patient-year-first analysis was not intended to replace the primary analysis, which preserves the cumulative burden reflected in repeated testing among clinically severe patients, but rather to serve as a sensitivity analysis to examine the extent to which repeated measurements influenced the estimates.

For clinical interpretation, CRP was dichotomized according to the predefined thresholds (≥1, ≥3, and ≥10 mg/dL). Frequencies and proportions were calculated by age group and sex, and Wilson 95% confidence intervals (CIs) were presented. For each threshold, logistic regression models including age group, sex, and the age group × sex interaction term were fitted to estimate odds ratios (ORs) and 95% CIs.

To account for within-patient correlation arising from repeated measurements, we additionally fitted logistic generalized estimating equation (GEE) models using the patient identifier as the clustering unit. As the aim of this study was to estimate population-averaged ORs rather than subject-specific effects, GEE was chosen over mixed-effects models. This approach allowed more robust estimation of regression coefficients and standard errors by accounting for correlation among repeated observations within the same patient, and reduced the possibility that statistical significance might be overstated simply because of the large number of test-level observations. An exchangeable working correlation structure and robust sandwich standard errors were used. All statistical tests were two-sided, and *p* < 0.05 was considered statistically significant. All analyses were performed using R version 4.3.3 (R Foundation for Statistical Computing, Vienna, Austria).

### 2.5. Ethical Considerations

This study was conducted in accordance with the ethical principles of the Declaration of Helsinki (1975; revised in 2013) and was approved by the Institutional Review Board (IRB) of Dankook University Hospital (approval number: DKUH IRB 2025-09-002; approval date: 9 September 2025). As this study involved a retrospective analysis of anonymized data without identifiable personal information, the requirement for informed consent was waived.

## 3. Results

### 3.1. Baseline Characteristics of the Test Records

**Figure 1:**
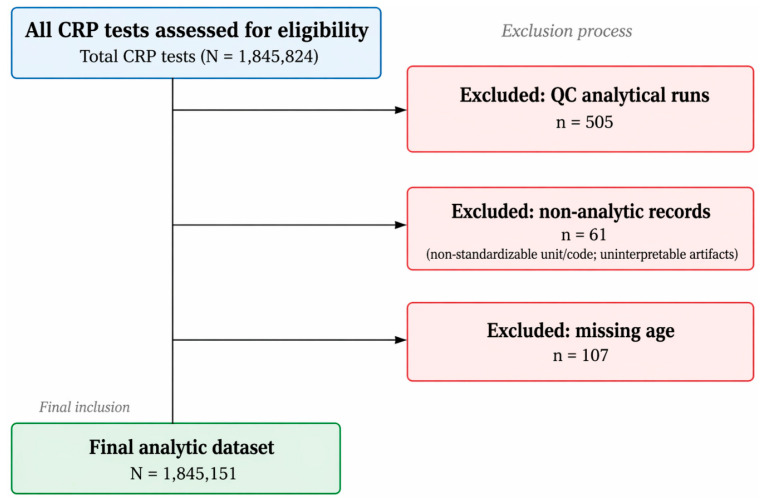
Flow diagram of CRP test record selection for the analytic dataset (2008–2023). All serum CRP test records performed at Dankook University Hospital (Cheonan, Republic of Korea) between 2008 and 2023 were assessed for eligibility (*N* = 1,845,824). We excluded 505 quality control (QC) results corresponding to non-patient analytical runs. A further 61 non-analytic records were removed because of non-standardizable units/codes or non-interpretable artifacts, and 107 records were excluded because age information was missing. The final analytic dataset comprised 1,845,151 CRP test records. The blue box indicates the full set of CRP test records assessed for eligibility, the red boxes indicate excluded records, and the green box indicates the final analytic dataset. The arrows represent the record selection process.

This study was based on serum CRP test records obtained at a single tertiary-care hospital, Dankook University Hospital (Cheonan, Republic of Korea), between 2008 and 2023. According to the record selection flowchart shown in Figure 1, 1,845,151 CRP test records were ultimately included in the analysis, representing 336,360 patients. The median age of the study population was 59.45 years (Q1–Q3, 42.52–73.12) (Table 1).

**Figure 2:**
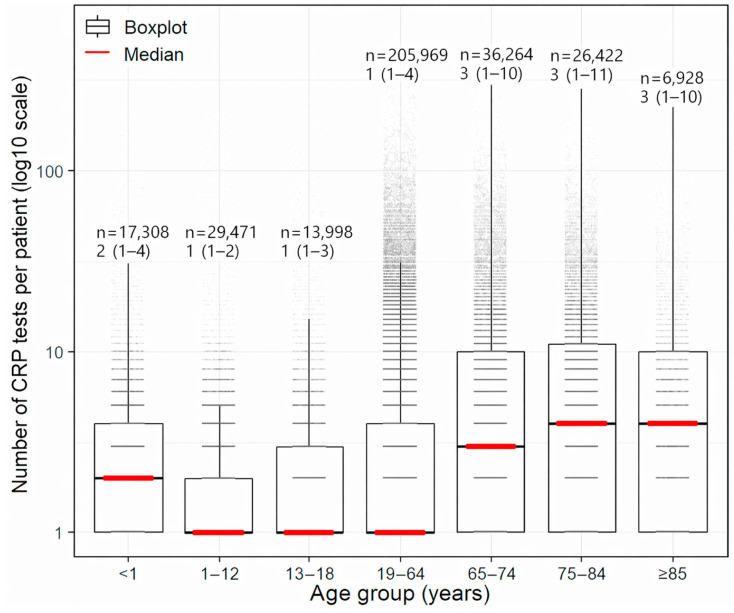
Age-stratified distribution of CRP testing intensity per patient. The y-axis shows the number of CRP tests per patient on a log10 scale. Boxplots indicate the interquartile range (IQR), with red lines denoting the median number of tests per patient in each age group; whiskers extend to 1.5 × IQR. Individual observations are overlaid to illustrate distributional density. Above each box, the patient count (*n*) and median (IQR) number of tests per patient are annotated.

The overall median CRP level was 0.79 mg/dL (Q1–Q3, 0.20–4.18) (Table 1). At the patient level, evaluation of the repeated-testing structure showed that the frequency of repeated CRP testing increased with age. The median number of CRP tests per patient (interquartile range) increased from 1 (1–4) in those aged 19–64 years to 3 (1–10) in those aged 65–74 years, and was even higher in those aged 75–84 years and ≥85 years, at 4 (1–11) and 4 (1–10), respectively. To more clearly illustrate the right-tailed nature of this distribution, the data were visualized using boxplots on a log10 scale (Figure 2).

This age-related heterogeneity in repeated testing suggests that test-level analyses may be influenced by patient composition and the structure of repeated measurements. Accordingly, in subsequent analyses, these potential effects were further evaluated using a patient-year-first sensitivity analysis and GEE models.

### 3.2. Age- and Sex-Specific CRP Distributions: Central Tendency and Upper-Tail Expansion

**Figure 3:**
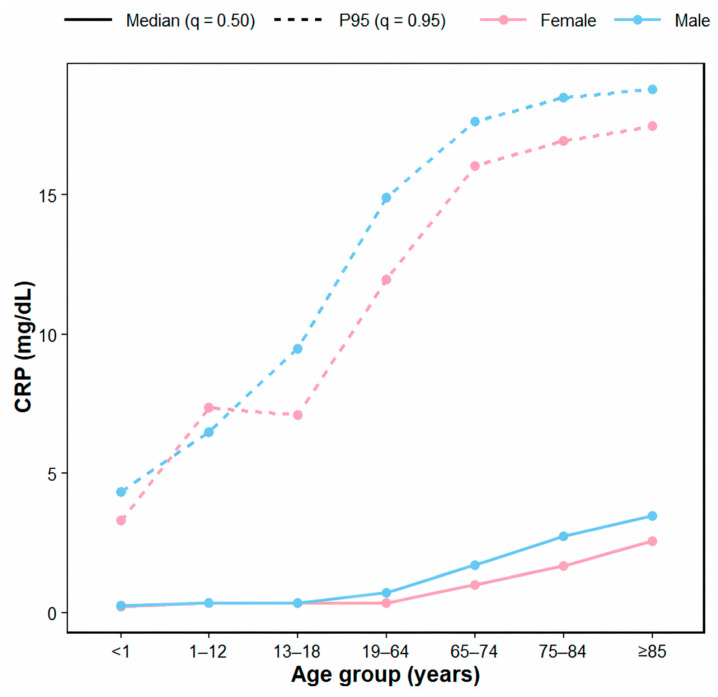
Age- and sex-specific trajectories of CRP central tendency and upper-tail reference levels. CRP concentrations (mg/dL) are shown across seven age groups, stratified by sex. Solid lines indicate the median (q = 0.50), and dashed lines indicate the 95th percentile (q = 0.95). With increasing age, the median and the upper-tail boundary shift upward, indicating that the distributional threshold for unusually high CRP values rises substantially across older age groups.

The median CRP level (q = 0.50) showed some fluctuations across the intermediate age groups but, overall, demonstrated a clear upward pattern in older adults. In the <1-year age group, the median was 0.19 mg/dL in females and 0.26 mg/dL in males; by ages 19–64 years, it had increased to 0.34 mg/dL in females and 0.69 mg/dL in males. The increase became more pronounced in the older age groups, reaching 0.98 and 1.70 mg/dL at ages 65–74 years, 1.66 and 2.70 mg/dL at ages 75–84 years, and 2.55 and 3.44 mg/dL at ages ≥ 85 years in females and males, respectively (Table 2; Figure 3).

The upper tail of the CRP distribution (q = 0.95, 95th percentile) also increased with age. In those aged <1 year, the 95th percentile was 3.28 mg/dL in females and 4.31 mg/dL in males, increasing to 12.00 and 14.90 mg/dL, respectively, in the 19–64-year group. In the older age groups, the 95th percentile remained high, with a further modest increase observed at ages 65–74 years (16.00 and 17.60 mg/dL), 75–84 years (16.90 and 18.50 mg/dL), and ≥85 years (17.50 and 18.80 mg/dL) in females and males, respectively (Table 2).

The effect of age group on the upper inflammatory burden (q = 0.95) was significant overall (all *p* < 0.001), and a sex effect and an age group × sex interaction were also observed (Table S1). In females and males, the 95th percentile increased markedly with age, and males generally showed higher values than females. However, the magnitude and statistical significance of this male predominance varied across age groups (Table S1).

These patterns were consistently reproduced in the trend analysis in which the age group was modeled as an ordinal score. The median (q = 0.50) and the 95th percentile (q = 0.95) increased significantly with each one-category increase in age group (both *p* < 0.001), and this age-related slope was additionally steeper in males (age × male interaction: β = 0.115 and β = 0.151, respectively; both *p* < 0.001). Although males showed overall higher levels at the 95th percentile (β = 1.633, *p* < 0.001), the main effect of sex at the reference age group was not significant for the median (β = −0.020, *p* = 0.0898) (Table S2).

### 3.3. Demographic Distribution of Elevated CRP (≥1, ≥3, and ≥10 mg/dL)

**Figure 4:**
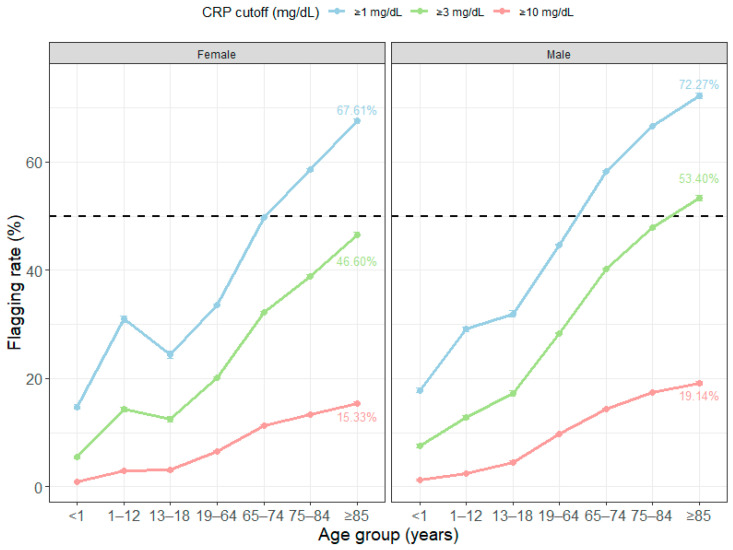
Age- and sex-specific prevalence of elevated C-reactive protein at three thresholds (≥1, ≥3, and ≥10 mg/dL). Line plots show the proportion of tests meeting each CRP threshold across seven age groups (<1, 1–12, 13–18, 19–64, 65–74, 75–84, and ≥85 years), stratified by sex (female, left; male, right). Lines represent CRP thresholds of ≥1 mg/dL (blue), ≥3 mg/dL (green), and ≥10 mg/dL (red). The horizontal dashed line indicates 50% prevalence. The figure highlights that lower thresholds become increasingly common with age, whereas higher thresholds also rise in parallel, especially in older men.

In the age- and sex-stratified analysis, the frequency of elevated CRP increased progressively with age and became particularly pronounced among adults aged ≥ 65 years (Figure 4). For CRP ≥ 1 mg/dL, the frequency in females approached one-half at ages 65–74 years (49.8%) and then increased further to 58.6% at ages 75–84 years and 67.6% at ages ≥ 85 years. In males, the frequency had already exceeded one-half at ages 65–74 years (58.3%) and increased further to 66.6% at ages 75–84 years and 72.3% at ages ≥ 85 years.

The same directional pattern was maintained at higher cutoff values. For CRP ≥ 3 mg/dL, the proportions were 32.2% in females and 40.3% in males at ages 65–74 years, 39.0% and 47.8% at ages 75–84 years, and 46.6% and 53.4% at ages ≥ 85 years. For CRP ≥ 10 mg/dL, the corresponding proportions were 11.2% and 14.4% at ages 65–74 years, 13.4% and 17.4% at ages 75–84 years, and 15.3% and 19.1% at ages ≥ 85 years in females and males, respectively (Table 3). Notably, among test records from individuals aged ≥ 85 years, the burden of elevated CRP was concentrated at all three thresholds: 67.6% in females and 72.3% in males for CRP ≥ 1 mg/dL, 46.6% and 53.4% for CRP ≥ 3 mg/dL, and 15.3% and 19.1% for CRP ≥ 10 mg/dL.

In models including the age group × sex interaction, the odds of elevated CRP increased significantly in the older age groups relative to the reference group (females aged < 1 year). For CRP ≥ 10 mg/dL, the ORs increased stepwise to 12.7 at ages 65–74 years, 15.4 at ages 75–84 years, and 18.1 at ages ≥ 85 years (Table S3). In the model for CRP ≥ 3 mg/dL, the age group × sex interaction was significant (χ^2^(6) = 752.8, *p* < 0.001), indicating that the magnitude of the male–female difference was not constant across age groups. To clarify the practical implications of these findings, representative age- and sex-specific examples illustrating context-dependent interpretation of common CRP values are provided in Table 4.

### 3.4. Sensitivity Analyses Accounting for Repeated Measurements

To mitigate potential bias related to the influence of patients with frequent repeated testing (i.e., testing intensity), we conducted a patient-year-first sensitivity analysis in which only the first CRP result from each patient in each calendar year was included. Overall, the principal patterns observed in the primary test-level analysis were preserved. Specifically, the median CRP level (q = 0.50) and the 95th percentile (q = 0.95) increased with age, and expansion of the upper-tail inflammatory burden remained evident in older age groups, particularly at the 95th percentile (Table S4). Although absolute CRP levels were generally lower than those in the primary analysis, the overall direction of the age-related upward shift in the distribution and upper-tail expansion remained unchanged. For example, the median increased from 0.11/0.15 mg/dL in females/males aged < 1 year to 0.53/0.71 mg/dL in those aged ≥ 85 years, whereas the 95th percentile increased from 3.40/4.39 mg/dL to 15.70/17.10 mg/dL, respectively (Table S4). In the quantile regression analysis, the effect of age remained significant for the median and the 95th percentile. Although some sex differences were observed for the median, these were neither as strong nor as consistent as those observed for the 95th percentile. By contrast, at the 95th percentile, the age-related slope was steeper in males (Tables S5 and S6).

To statistically account for within-patient correlation arising from repeated measurements, we additionally performed GEE logistic regression analyses for each CRP cutoff (≥1, ≥3, and ≥10 mg/dL), including the age group × sex interaction term. The stepwise increase in the risk of elevated CRP with advancing age was reproduced in the same direction as in the primary analysis. In particular, for CRP ≥10 mg/dL, the odds were significantly increased in the 65–74-year group (OR 12.5), 75–84-year group (OR 15.2), and ≥85-year group (OR 17.7) relative to the reference group (females aged < 1 year) (Table S7). Effect modification of sex across age groups was not fully consistent across cutoffs. For CRP ≥ 1 mg/dL, significant interactions were observed in the 1–12-year group (OR 0.736) and the 19–64-year group (OR 1.29), whereas no significant interaction was observed in the ≥85-year group (OR 1.01, *p* = 0.851). Similarly, for CRP ≥ 3 mg/dL, the magnitude of the interaction generally appeared to attenuate in the older age groups from 65 to 74 years onward (Table S7).

## 4. Discussion

This study shows that the same CRP threshold may not carry the same clinical weight across age and sex groups in hospital-based care. In this large real-world tertiary-care dataset, the overall distribution of CRP and the frequency of threshold exceedance varied substantially by age and sex, indicating that identical CRP values may represent different levels of concern depending on demographic context. Such patterns may be interpreted, in part, in light of inflammaging and the accumulation of comorbid conditions in older adults [15,16]. At the same time, because the present dataset did not include detailed linked clinical information, the observed age-stratified differences are likely to reflect not only demographic structure but also differences in healthcare utilization and hospital-based case-mix. Therefore, when fixed thresholds developed for general use are applied uniformly across age groups, their interpretive weight may differ according to demographic and clinical context [17,18]. Our findings support the need for a contextualized approach to CRP interpretation that considers not only the absolute value itself but also the position of that value within the age- and sex-specific distribution.

The starting point for this interpretation is the marked age-related difference in testing intensity. The fact that older groups underwent more repeated testing per patient and showed a strongly right-skewed distribution indicates that age comparisons in hospital-based laboratory data cannot be assumed to reflect biological differences alone [19]. The clinical context of older adults—including multimorbidity, hospitalization, complications, and follow-up testing—may thicken the distribution through repeated measurements and thereby increase the observed frequency of values exceeding a given threshold. Accordingly, describing the repeated-testing structure up front and explicitly designing the subsequent age comparisons to account for case-mix and heterogeneity in repeated measurements was a key feature that strengthened the interpretability of the results [20].

Considering the median together with the 95th percentile (upper tail) provides a clinically more meaningful quantification of the age effect on CRP [21]. Previous studies have reported that CRP tends to increase with age [22]. In the present study, however, the more important finding is that this increase was accompanied by expansion of the upper tail, indicating a change in the shape of the distribution rather than a simple upward shift in location. Clinical decision-making is often driven less by the mean or median than by exceptionally high values. The observation that the 95th percentile remained high and continued to rise in older adults suggests not simply that CRP is slightly higher overall, but that the subgroup carrying a high inflammatory burden becomes larger with age. More importantly, this implies that the distributional boundary for what may be considered unusually high CRP is itself shifted upward in older populations. A value located in the upper tail among younger individuals may therefore be less exceptional in older adults, and a single threshold applied without regard to age cannot capture this distributional displacement. Sex differences were also more evident in the upper tail than at the median, and their magnitude and direction varied across age groups. Interpretation of the <1 year group also requires caution because this category includes the neonatal and early postnatal period, during which CRP may follow a physiological pattern distinct from that in later infancy or adulthood. In particular, in the 1–12-year age group, a negative interaction was observed, with the male 95th percentile lower than that in females, unlike the pattern seen in other age groups. This may reflect subtle sex-related differences in innate immunity and inflammatory responses in childhood, differences in healthcare utilization and testing indications, or sex differences in the distribution of specific diagnostic categories within the upper tail. Although the present dataset did not include diagnosis or clinical context and thus cannot directly elucidate the mechanism, this exception nevertheless suggests that the effect of sex on CRP does not operate uniformly across all age groups.

The threshold-based findings are not easily explained by a simple increase in baseline inflammation alone. The key clinical insight of this study is that, in the older population of this tertiary-care testing cohort, backgrounding of mild elevation and concomitant increases in marked elevation occurred simultaneously. In particular, in the ≥85-year age group, CRP ≥ 1 mg/dL was observed in 67.6% of females and 72.3% of males, whereas CRP ≥ 10 mg/dL was present in 15.3% and 19.1%, respectively, indicating that a high inflammatory burden is far from exceptional in this age group [23]. Among individuals aged ≥65 years, CRP ≥ 1 mg/dL was already present in approximately one-half or more, and the proportion rose further with advancing age. However, this shift was not limited to mild elevation. At higher thresholds, including ≥3 mg/dL and ≥10 mg/dL, the proportions also increased clearly with age. In those aged ≥85 years, CRP ≥ 3 mg/dL approached 50% of the group, and CRP ≥ 10 mg/dL was observed at a level that is not clinically negligible. Thus, in later life, mild elevation becomes a common background state, whereas marked elevation—potentially associated with acute deterioration, infection, or tissue injury—also increases in parallel. This combination suggests that the clinical context surrounding a given threshold may differ across age groups, even when the same conventional cutoff is retained. A CRP value of 1 mg/dL may reflect chronic inflammatory background or comorbidity burden in many patients, whereas a value of ≥10 mg/dL may still remain strongly relevant to acute worsening, infection requiring antimicrobial treatment, or tissue injury [24,25,26]. Ultimately, the central clinical implication of this study is that the same numeric value may carry a different clinical weight depending on age group. For example, a CRP value of approximately 1 mg/dL may represent a relatively uncommon inflammatory signal in younger individuals, whereas in the oldest-old hospital-based population it may fall within a much more common background range. By contrast, a CRP value of 10 mg/dL remains clinically important across age groups, but in older adults it should be interpreted with awareness that baseline frequency and underlying clinical complexity are higher.

These findings are consistent with reports from international population studies showing that CRP distributions depend on sex and age [7,27]. However, the contribution of the present study is not simply another confirmation that CRP increases with age. Rather, using long-term hospital-based real-world data, we quantified how the frequencies of clinically familiar thresholds—1, 3, and 10 mg/dL—are reconfigured across age groups, and showed that this change occurs not only through a shift in central tendency but also through expansion of the upper tail. Even when the same threshold is used, if the frequency with which that threshold occurs differs by age group, then the clinical meaning of the same “positive” or “elevated” classification will inevitably differ as well.

A major strength of this study lies in its explicit handling of the repeated-measurement structure. Importantly, the two sensitivity approaches addressed different aspects of repeated testing. The patient-year-first analysis was designed to reduce the possibility that repeated tests from a subset of patients, driven by differences in testing intensity across age groups, might disproportionately shape the overall distribution [28]. Although absolute CRP levels became somewhat lower under this approach, the direction of the age-related upward shift and upper-tail expansion remained unchanged. By contrast, the GEE logistic analysis statistically accounted for within-patient correlation arising from repeated measurements [29]. Even under this framework, the stepwise increase in the risk of elevated CRP with age was reproduced in the same direction. This suggests that the higher threshold frequencies observed in older adults are not merely artificial products of more frequent testing in a high-utilization subgroup, but rather relatively robust signals that persist even after the correlation structure is considered. The observation that sex differences were more pronounced in the upper tail than at the median, and that this directionality persisted even in analyses that attenuated the impact of repeated testing, further suggests that the observed sex differences may reflect heterogeneity in the formation of upper inflammatory burden rather than being simple by-products of the repeated-measurement structure.

The clinical implication is not that existing CRP thresholds should be replaced but that their interpretation may benefit from consideration of age- and sex-specific distributional context in hospital-based populations. In younger individuals, a value of ≥1 mg/dL may represent a relatively uncommon signal, whereas in older adults within this hospital-based cohort, it may constitute a common background state. Conversely, the fact that CRP ≥10 mg/dL becomes more frequent in old age should not lead to the conclusion that it is therefore less important. Rather, its reasonable prevalence within that age group should itself be recognized as a structural signal linked to healthcare resource use, disease severity, and the burden of differentiating infectious from non-infectious causes. In practice, it may be safer to retain absolute-value interpretation of CRP while incorporating age- and sex-specific relative position—for example, how close a value such as 10 mg/dL is to the upper percentiles within the relevant demographic group—together with the repeated-measurement context, such as whether the value represents an initial measurement or one obtained during follow-up. The strengths of this study include its large-scale, long-term real-world laboratory dataset spanning 16 years and its simultaneous characterization of distributional change and reconfiguration of threshold frequencies according to age and sex. By describing the repeated-testing structure and examining robustness through sensitivity analyses and GEE models, we addressed a major source of bias inherent to hospital-based data.

Nevertheless, some limitations should be acknowledged. As the data were derived from a single tertiary-care hospital, the results reflect regional and healthcare-utilization-related case-mix, and should not be directly extended as reference intervals for the general population [30]. Older age groups may have included a disproportionately higher number of inpatients, frail individuals, multimorbid patients, and episodes involving acute deterioration or emergency care, such that part of the observed age effect may reflect this clinical composition as well as biological aging itself. In addition, because we were unable to fully incorporate clinical covariates that may influence CRP, such as diagnosis, comorbidity, obesity, smoking, medication use, or care setting (outpatient, inpatient, or emergency), the observed age- and sex-related differences cannot be decomposed into specific mechanisms. Accordingly, the present findings are best interpreted as distributional patterns observed within a tertiary-care hospital population, in which age-related differences may reflect demographic variation and the greater clinical complexity often accompanying older age. This consideration may be particularly relevant for higher CRP thresholds, for which observed frequencies may also reflect differences in the burden of active inflammatory conditions across age strata within a hospital-based cohort. As the analysis was conducted at the test-record level, linkage to patient-level clinical outcomes such as hospitalization, intensive care unit admission, antimicrobial use, or mortality will require further study.

Nonetheless, despite these limitations, the present study simultaneously evaluated the center (median) and upper tail (95th percentile) of the CRP distribution using a large real-world dataset accumulated over 16 years, and further strengthened the consistency and robustness of the findings through patient-year-first sensitivity analysis and GEE-based correction for distortion related to repeated testing. Given that the aim of this study was not to determine the etiology of a specific disease or to predict clinical prognosis, but rather to re-examine the distributional assumptions underlying fixed-cutoff interpretation in age- and sex-varying CRP distributions, these limitations may constrain the scope of external validity but do not fundamentally undermine the main conclusions.

Future studies should proceed in several directions. First, the same analytical framework—combining the median, upper tail, threshold frequencies, and adjustment for repeated measurements—should be replicated in multicenter datasets to establish external validity. Second, linkage with electronic medical records should be used to stratify how the meaning of given thresholds varies by diagnostic category (infectious vs. non-infectious), comorbidity burden, and clinical setting (emergency department, inpatient, or outpatient), and to determine, in older adults, to what extent CRP ≥ 1 mg/dL reflects chronic inflammatory background and how strongly CRP ≥ 10 mg/dL is associated with acute deterioration on an event basis, such as hospitalization, initiation of antimicrobial therapy, or ICU admission. Third, for clinical implementation, percentile-based reporting by age and sex—such as simultaneous provision of p50 and p95 for each demographic group—and risk-based interpretation algorithms that integrate age, sex, repeated-measurement trajectory, and clinical context should be developed and prospectively evaluated for their ability to improve appropriateness of decision-making, including reduction in unnecessary antibiotics, avoidable hospitalization, and diagnostic misclassification.

Taken together, CRP should not be interpreted as a single number that uniformly defines a patient, but rather as a biomarker that must be understood within its distributional context. In an aging healthcare environment, CRP ≥ 1 mg/dL becomes a common background state, whereas more marked elevations of ≥3 and ≥10 mg/dL also increase meaningfully, so that the clinical center of gravity of the same threshold differs according to age and sex. An interpretive approach that integrates demographic context and repeated-measurement structure into CRP assessment may provide a practical basis for reducing under-recognition of meaningful risk signals and overinterpretation of boundary values, thereby improving the precision of clinical decision-making.

## 5. Conclusions

This study provides quantitative hospital-based evidence, based on large-scale real-world laboratory data from 2008 to 2023, that the observed CRP distribution differs substantially across age and sex strata not only in its central tendency but also in the upper tail, such that the same numeric value may carry different distributional meaning across age groups. In older age strata within this tertiary-care cohort, common mild elevation (≥1 mg/dL) coexisted with a higher proportion of marked elevation (≥10 mg/dL), suggesting that the interpretive implications of fixed CRP thresholds may not be uniform across age groups. Accordingly, fixed thresholds may still remain practical clinical tools, but their interpretation should be refined by incorporating information on relative position within the age- and sex-specific distribution as well as threshold frequency. These data may serve as a descriptive reference for understanding how conventional CRP thresholds are distributed across age and sex strata in hospital-based practice. In clinical settings, this approach may help reduce overinterpretation of mild elevations that become common in older adults, while preserving the value of marked elevations as signals to support severity assessment, differential diagnosis, and more precise resource allocation.

## Figures and Tables

**Table 1:** Baseline characteristics of CRP test records (2008–2023).

Characteristic	Value
Total test records, *n*	1,845,151
Patients, *n*	336,360
Sex (test-level), *n* (%)	Male 1,074,449 (58.2); Female 770,702 (41.8)
Age, years	Mean ± SD: 55.44 ± 22.51; Median (IQR): 59.45 (42.52–73.12); Range: 0–112
Age group (test-level), *n* (%)	<1: 51,727 (2.80); 1–12: 64,165 (3.48); 13–18: 39,721 (2.15); 19–64: 958,043 (51.9); 65–74: 340,655 (18.5); 75–84: 309,297 (16.8); ≥85: 81,543 (4.42)
CRP (mg/dL), *n*	1,845,151
CRP (mg/dL)	Mean ± SD: 3.37 ± 5.60; Median (IQR): 0.79 (0.20–4.18); Range: 0.03–69.1

**Table 2:** Test-level analysis of CRP distribution by age group and sex (median and 95th percentile).

Age Group (Years)	Sex	Tests, n	Median (q = 0.50), mg/dL	95th Percentile (q = 0.95), mg/dL
<1	F	21,724	0.19	3.28
<1	M	30,003	0.26	4.31
1–12	F	27,296	0.34	7.31
1–12	M	36,869	0.34	6.47
13–18	F	15,181	0.32	7.09
13–18	M	24,540	0.34	9.47
19–64	F	368,733	0.34	12.00
19–64	M	589,310	0.69	14.90
65–74	F	139,967	0.98	16.00
65–74	M	200,688	1.70	17.60
75–84	F	149,985	1.66	16.90
75–84	M	159,312	2.70	18.50
≥85	F	47,816	2.55	17.50
≥85	M	33,727	3.44	18.80

**Table 3:** Age- and sex-stratified prevalence of elevated CRP (≥1, ≥3, and ≥10 mg/dL) with Wilson 95% confidence intervals.

Age Group	Sex	Cutoff	n	x	Rate (%)	95% CI (Wilson), %
<1	F	≥1	21,724	3211	14.8	14.3–15.3
	F	≥3	21,724	1182	5.44	5.15–5.75
	F	≥10	21,724	215	0.99	0.866–1.13
	M	≥1	30,003	5352	17.8	17.4–18.3
	M	≥3	30,003	2267	7.56	7.26–7.86
	M	≥10	30,003	391	1.30	1.18–1.44
1–12	F	≥1	27,296	8462	31.0	30.5–31.6
	F	≥3	27,296	3902	14.3	13.9–14.7
	F	≥10	27,296	817	2.99	2.80–3.20
	M	≥1	36,869	10,729	29.1	28.6–29.6
	M	≥3	36,869	4732	12.8	12.5–13.2
	M	≥10	36,869	911	2.47	2.32–2.63
13–18	F	≥1	15,181	3704	24.4	23.7–25.1
	F	≥3	15,181	1890	12.4	11.9–13.0
	F	≥10	15,181	468	3.08	2.82–3.37
	M	≥1	24,540	7841	32.0	31.4–32.5
	M	≥3	24,540	4239	17.3	16.8–17.8
	M	≥10	24,540	1111	4.53	4.27–4.79
19–64	F	≥1	368,733	123,809	33.6	33.4–33.7
	F	≥3	368,733	74,263	20.1	20.0–20.3
	F	≥10	368,733	24,311	6.59	6.51–6.67
	M	≥1	589,310	263,537	44.7	44.6–44.8
	M	≥3	589,310	167,380	28.4	28.3–28.5
	M	≥10	589,310	57,466	9.75	9.68–9.83
65–74	F	≥1	139,967	69,639	49.8	49.5–50.0
	F	≥3	139,967	45,112	32.2	32.0–32.5
	F	≥10	139,967	15,743	11.2	11.1–11.4
	M	≥1	200,688	117,037	58.3	58.1–58.5
	M	≥3	200,688	80,782	40.3	40.0–40.5
	M	≥10	200,688	28,996	14.4	14.3–14.6
75–84	F	≥1	149,985	87,836	58.6	58.3–58.8
	F	≥3	149,985	58,448	39.0	38.7–39.2
	F	≥10	149,985	20,024	13.4	13.2–13.5
	M	≥1	159,312	106,180	66.6	66.4–66.9
	M	≥3	159,312	76,230	47.8	47.6–48.1
	M	≥10	159,312	27,741	17.4	17.2–17.6
≥85	F	≥1	47,816	32,328	67.6	67.2–68.0
	F	≥3	47,816	22,282	46.6	46.2–47.0
	F	≥10	47,816	7328	15.3	15.0–15.7
	M	≥1	33,727	24,374	72.3	71.8–72.7
	M	≥3	33,727	18,011	53.4	52.9–53.9
	M	≥10	33,727	6456	19.1	18.7–19.6

For each age group and sex stratum, n denotes the total number of CRP tests and x denotes the number of tests meeting the specified threshold; rates are presented as percentages with Wilson 95% confidence intervals.

**Table 4:** Representative age- and sex-specific examples for contextual interpretation of common CRP values in hospital-based practice.

Demographic Group	Example CRP Value	Median CRP (mg/dL)	95th Percentile (mg/dL)	Relevant Frequency in that Group	Example of Contextual Interpretation
Female, <1 year	1 mg/dL	0.19	3.28	CRP ≥ 1 mg/dL: 14.8%	In this group, 1 mg/dL is above the median and not highly prevalent; it may therefore represent a more meaningful inflammatory signal when interpreted with symptoms and other clinical findings.
Male, 19–64 years	1 mg/dL	0.69	14.90	CRP ≥ 1 mg/dL: 44.7%	In hospital-based adult males, 1 mg/dL is only modestly above the median and relatively common; isolated interpretation should therefore be cautious.
Female, ≥85 years	1 mg/dL	2.55	17.50	CRP ≥ 1 mg/dL: 67.6%	In the oldest-old female group, 1 mg/dL may fall within a common background inflammatory range and should not be overinterpreted in isolation.
Female, ≥85 years	3 mg/dL	2.55	17.50	CRP ≥ 3 mg/dL: 46.6%	A value of approximately 3 mg/dL is relatively frequent in this group and should be interpreted in demographic and clinical context rather than treated as uniformly alarming in isolation.
Male, ≥85 years	10 mg/dL	3.44	18.80	CRP ≥ 10 mg/dL: 19.1%	Although more frequent than in younger groups, 10 mg/dL remains a marked elevation and should prompt evaluation for acute infection, tissue injury, or clinical deterioration.

These examples are intended to translate the observed age- and sex-specific CRP distributions into a clinically interpretable framework, illustrating that the same CRP value may carry different inflammatory significance depending on demographic context in this hospital-based cohort. As such, they may support more nuanced interpretation of CRP results alongside symptoms, comorbidities, and clinical trajectory, while complementing overall clinical judgment.

## Data Availability

The data underlying the results of this study originate from clinical records at Dankook University Hospital and are governed by ethical and legal regulations. To protect patient privacy and confidentiality, the original datasets are not publicly accessible. Nevertheless, de-identified aggregated data may be provided by the corresponding author upon reasonable request, contingent on approval from the Institutional Review Board.

## Supplementary Materials

The following supporting information can be downloaded at: https://www.mdpi.com/article/10.3390/diagnostics16091268/s1, Table S1: Quantile regression for the upper tail of CRP (τ = 0.95): age group × sex interaction; Table S2: Ordinal age-trend quantile regression of CRP at the median and 95th percentile (τ = 0.50 and τ = 0.95); Table S3: Odds ratios for elevated CRP at three thresholds (≥1, ≥3, and ≥10 mg/dL) from logistic regression models including age group, sex, and age-by-sex interaction; Table S4: Patient-year–level CRP distribution by age group and sex (first CRP per patient-year); Table S5: Quantile regression for median CRP (q = 0.50) and upper-tail CRP (q = 0.95): age group × sex interaction, patient-year first analysis; Table S6: Ordinal age-group trend models for median and 95th-percentile CRP, patient-year first analysis; Table S7: Logistic GEE for elevated CRP (≥1, ≥3, and ≥10 mg/dL).

## Abbreviations

The following abbreviations are used in this manuscript:

CRP	C-reactive protein
QC	Quality control
GEE	Generalized estimating equations
OR	Odds ratio
CI	Confidence interval
LIS	Laboratory information system
