# Supplementary material for: Age- and Sex-Dependent Interpretation of C-Reactive Protein Cutoffs: A Sixteen-Year Large-Scale Clinical Laboratory Data Analysis
Source: Diagnostics (Basel). 2026 Apr 23;16(9):1268. doi: 10.3390/diagnostics16091268 (PMC13163134; doi:10.3390/diagnostics16091268)
Supplement: Supplementary file 1 [file diagnostics-16-01268-s001.zip › diagnostics-4232654-supplementary.pdf]

Supplementary Tables

Table S1. Quantile regression for the upper tail of CRP ($\tau = 0.95$): age group × sex interaction

**Term**	**Estimate (β), mg/dL**	**p-value**
(Intercept)	3.28	<0.001
Age group 1–12	4.03	<0.001
Age group 13–18	3.81	<0.001
Age group 19–64	8.67	<0.001
Age group 65–74	12.77	<0.001
Age group 75–84	13.64	<0.001
Age group ≥85	14.22	<0.001
Male (vs Female)	1.03	<0.001
1–12 × Male	−1.87	<0.001
13–18 × Male	1.36	<0.001
19–64 × Male	1.90	<0.001
65–74 × Male	0.54	0.00191
75–84 × Male	0.53	0.00186
≥85 × Male	0.26	0.282

Estimates represent differences in the conditional 95th percentile of CRP relative to the reference group (<1-year females). Interaction terms indicate age-specific modification of the male effect; the male–female difference within each age group is calculated as Male + (Age group × Male).

Table S2. Ordinal age-trend quantile regression of CRP at the median and 95th percentile (τ = 0.50 and τ = 0.95)

Term	τ = 0.50 (Median) β, mg/dL	p-value	τ = 0.95 (95th percentile) β, mg/dL	p-value
(Intercept)	−0.340	<0.001	1.785	<0.001
age_ord (per category)	0.235	<0.001	2.555	<0.001
Male (vs Female)	−0.020	0.0898	1.633	<0.001
age_ord × Male	0.115	<0.001	0.151	<0.001

The age_ord coefficient represents the per-step change in the conditional CRP quantile across ordered age categories. The interaction term (age_ord × Male) indicates sex-specific differences in the age gradient (i.e., the additional per-step change in males relative to females).

Table S3. Odds ratios for elevated CRP at three thresholds (≥1, ≥3, and ≥10 mg/dL) from logistic regression models including age group, sex, and age-by-sex interaction

CRP cutoff	Term	OR	95% CI	p-value
≥1 mg/dL	(Intercept)	0.173	0.167–0.180	<0.001
	age_grp 1–12	2.59	2.48–2.71	<0.001
	age_grp 13–18	1.86	1.77–1.96	<0.001
	age_grp 19–64	2.91	2.81–3.03	<0.001
	age_grp 65–74	5.71	5.49–5.94	<0.001
	age_grp 75–84	8.15	7.84–8.47	<0.001
	age_grp ≥85	12	11.5–12.6	<0.001
	sex Male	1.25	1.19–1.31	<0.001
	1–12 × Male	0.73	0.688–0.774	<0.001
	13–18 × Male	1.16	1.09–1.24	<0.001
	19–64 × Male	1.28	1.22–1.34	<0.001
	65–74 × Male	1.13	1.07–1.19	<0.001
	75–84 × Male	1.13	1.07–1.19	<0.001
	≥85 × Male	0.997	0.942–1.06	0.929
≥3 mg/dL	(Intercept)	0.0575	0.0542–0.0610	<0.001
	age_grp 1–12	2.9	2.71–3.10	<0.001
	age_grp 13–18	2.47	2.29–2.67	<0.001
	age_grp 19–64	4.38	4.13–4.65	<0.001
	age_grp 65–74	8.27	7.79–8.78	<0.001
	age_grp 75–84	11.1	10.5–11.8	<0.001
	age_grp ≥85	15.2	14.3–16.1	<0.001
	sex Male	1.42	1.32–1.53	<0.001
	1–12 × Male	0.621	0.570–0.677	<0.001
	13–18 × Male	1.03	0.942–1.13	0.485
	19–64 × Male	1.11	1.03–1.19	0.006
	65–74 × Male	0.997	0.926–1.07	0.942
	75–84 × Male	1.01	0.939–1.09	0.760
	≥85 × Male	0.925	0.855–0.999	0.048
≥10 mg/dL	(Intercept)	0.01	0.00871–0.0114	<0.001
	age_grp 1–12	3.09	2.66–3.60	<0.001
	age_grp 13–18	3.18	2.71–3.75	<0.001
	age_grp 19–64	7.06	6.19–8.11	<0.001
	age_grp 65–74	12.7	11.1–14.6	<0.001
	age_grp 75–84	15.4	13.5–17.7	<0.001
	age_grp ≥85	18.1	15.8–20.8	<0.001
	sex Male	1.32	1.12–1.56	0.001
	1–12 × Male	0.622	0.512–0.753	<0.001
	13–18 × Male	1.13	0.923–1.38	0.236
	19–64 × Male	1.16	0.978–1.37	0.085
	65–74 × Male	1.01	0.851–1.19	0.918
	75–84 × Male	1.04	0.874–1.22	0.680
	≥85 × Male	0.99	0.833–1.17	0.910

Odds ratios were estimated from logistic regression models including age group, sex, and age-by-sex interaction, with females aged <1 year as the reference group. Thus, the main effect of age group represents the age effect among females, the main effect of sex represents the male effect in the <1-year age group, and the male effect within each age group is obtained by combining the coefficient for Male with the corresponding age group × Male interaction term.

Table S4. Patient-year–level CRP distribution by age group and sex (first CRP per patient-year)

Age group (years)	Sex	Patient-years, n	Median (q=0.50), mg/dL	95th percentile (q=0.95), mg/dL
<1	F	7,930	0.11	3.40
<1	M	10,550	0.15	4.39
1–12	F	16,807	0.33	5.86
1–12	M	22,359	0.32	5.34
13–18	F	7,751	0.25	4.01
13–18	M	10,351	0.31	4.53
19–64	F	138,155	0.25	4.94
19–64	M	162,185	0.26	6.57
65–74	F	30,608	0.26	9.77
65–74	M	36,140	0.31	11.60
75–84	F	26,770	0.31	13.00
75–84	M	24,850	0.35	14.90
≥85	F	8,153	0.53	15.70
≥85	M	5,207	0.71	17.10

Table S5. Quantile regression for median CRP (q=0.50) and upper-tail CRP (q=0.95): age group × sex interaction, patient-year first analysis

Term	q=0.50 Estimate (β), mg/dL	SE	p-value	q=0.95 Estimate (β), mg/dL	SE	p-value
(Intercept)	0.11	0.00461	<0.001	3.4	0.115	<0.001
Age group 1–12	0.22	0.00488	<0.001	2.46	0.16855	<0.001
Age group 13–18	0.14	0.01696	<0.001	0.61	0.22674	0.007
Age group 19–64	0.14	0.00511	<0.001	1.54	0.13104	<0.001
Age group 65–74	0.15	0.00746	<0.001	6.37	0.21582	<0.001
Age group 75–84	0.2	0.00478	<0.001	9.56	0.24631	<0.001
Age group ≥85	0.42	0.02098	<0.001	12.31	0.38504	<0.001
Male	0.04	0.00756	<0.001	0.99	0.18395	<0.001
1–12 × Male	−0.05	0.00785	<0.001	−1.51	0.24021	<0.001
13–18 × Male	0.02	0.0181	0.269	−0.47	0.32158	0.144
19–64 × Male	−0.03	0.00828	<0.001	0.64	0.20666	0.002
65–74 × Male	0.01	0.00963	0.299	0.89	0.33298	0.008
75–84 × Male	0	0.00861	1	0.92	0.36711	0.012
≥85 × Male	0.14	0.04294	0.001	0.39	0.72867	0.593

Table S6. Ordinal age-group trend models for median and 95th-percentile CRP, patient-year first analysis

Quantile	Term	Estimate (β), mg/dL	SE	p-value
q=0.50	Intercept	0.22667	0.0049	<0.001
q=0.50	Age ordinal score	0.01333	0.0009	<0.001
q=0.50	Male	0.02083	0.00827	0.012
q=0.50	Age ordinal × Male	−0.00083	0.00183	0.649
q=0.95	Intercept	0.6425	0.13786	<0.001
q=0.95	Age ordinal score	1.5575	0.03779	<0.001
q=0.95	Male	0.2555	0.18727	0.172
q=0.95	Age ordinal × Male	0.2445	0.05186	<0.001

Coefficients represent the linear change in the conditional median (q=0.50) or 95th percentile (q=0.95) across ordered age groups. The interaction term (Age ordinal × Male) indicates whether the age-related slope differs by sex.

Table S7. Logistic GEE for elevated CRP (≥1, ≥3, and ≥10 mg/dL)

Cutoff	Category	Term	OR	95% CI	p-value
≥1 mg/dL	Age-group effects (Females; ref = <1 year females)	1–12 vs <1	2.54	2.43–2.66	<0.001
		13–18 vs <1	1.82	1.73–1.92	<0.001
		19–64 vs <1	2.86	2.75–2.97	<0.001
		65–74 vs <1	5.61	5.40–5.83	<0.001
		75–84 vs <1	8.01	7.70–8.33	<0.001
		≥85 vs <1	11.7	11.2–12.2	<0.001
	Sex effect (<1 year)	Male vs Female	1.24	1.19–1.30	<0.001
	Age×sex interaction (modification of male effect)	1–12 × Male	0.736	0.69–0.78	<0.001
		13–18 × Male	1.17	1.10–1.25	<0.001
		19–64 × Male	1.29	1.23–1.35	<0.001
		65–74 × Male	1.13	1.08–1.19	<0.001
		75–84 × Male	1.14	1.08–1.20	<0.001
		≥85 × Male	1.01	0.95–1.06	0.851
≥3 mg/dL	Age-group effects (Females; ref = <1 year females)	1–12 vs <1	2.84	2.65–3.04	<0.001
		13–18 vs <1	2.42	2.24–2.61	<0.001
		19–64 vs <1	4.30	4.05–4.56	<0.001
		65–74 vs <1	8.12	7.65–8.62	<0.001
		75–84 vs <1	10.9	10.3–11.6	<0.001
		≥85 vs <1	14.8	13.9–15.7	<0.001
	Sex effect (<1 year)	Male vs Female	1.41	1.31–1.51	<0.001
	Age×sex interaction (modification of male effect)	1–12 × Male	0.629	0.58–0.69	<0.001
		13–18 × Male	1.04	0.95–1.14	0.402
		19–64 × Male	1.12	1.04–1.20	0.003
		65–74 × Male	1.00	0.93–1.08	0.927
		75–84 × Male	1.02	0.95–1.10	0.592
		≥85 × Male	0.932	0.86–1.01	0.075
≥10 mg/dL	Age-group effects (Females; ref = <1 year females)	1–12 vs <1	3.00	2.58–3.50	<0.001
		13–18 vs <1	3.11	2.64–3.67	<0.001
		19–64 vs <1	6.94	6.06–7.94	<0.001
		65–74 vs <1	12.5	10.9–14.3	<0.001
		75–84 vs <1	15.2	13.2–17.4	<0.001
		≥85 vs <1	17.7	15.4–20.3	<0.001
	Sex effect (<1 year)	Male vs Female	1.31	1.11–1.55	0.001
	Age×sex interaction (modification of male effect)	1–12 × Male	0.627	0.52–0.76	<0.001

Cutoff	Category	Term	OR	95% CI	p-value
		13–18 × Male	1.13	0.93–1.39	0.220
		19–64 × Male	1.17	0.99–1.38	0.072
		65–74 × Male	1.01	0.86–1.20	0.894
		75–84 × Male	1.04	0.88–1.23	0.620
		≥85 × Male	0.994	0.84–1.18	0.946
